# Antioxidant and vasorelaxant activities induced by northeastern Brazilian fermented grape skins

**DOI:** 10.1186/s12906-017-1881-2

**Published:** 2017-07-28

**Authors:** José George F. Albuquerque, Valéria L. Assis, Arthur J. P. O. Almeida, Ionaldo J. L. D. Basílio, Melissa N. Luciano, Bruno R. L. A. Meireles, Ângela M. T. M. Cordeiro, Islânia G. A. Araújo, Robson C. Veras, Thaís P. Ribeiro, Isac A. Medeiros

**Affiliations:** 1 Universidade Federal da Paraíba, Instituto de Pesquisa em Fármacos e Medicamentos-IPeFarM - Campus I. Cidade Universitária, CEP 58051-970, João Pessoa, PB Brazil; 2 Universidade Federal da Paraíba, Laboratório de Análise Química de Alimentos. Cidade Universitária, CEP 58051-900, João Pessoa, PB Brazil; 3 Universidade Federal da Paraíba, Centro de Tecnologia e Desenvolvimento Regional. Mangabeira, CEP 58055-000, João Pessoa, PB Brazil; 4 Universidade Federal de Santa Catarina, UFSC - Campus Araranguá. Jardim das Avenidas, CEP 88906-072, Araranguá, SC Brazil

**Keywords:** Grape pomace, Antioxidant, Vasorelaxation

## Abstract

**Background:**

In northeastern Brazil, grape pomace has become a potential alternative byproduct because of the recover phenolic compounds from the vinification process. Comparative analyses were performed between lyophilized extract of grape skins from pomace, described as fermented (FGS), and fresh, unfermented (UGS) grape skins to show the relevant brand’s composition upon the first maceration in winemaking.

**Methods:**

The use of in vitro testing such as Folin-Ciocalteu’s, DPPH free radical scavenger and HPLC methods were performed to evidence antioxidant effect and phenolic compounds. Additionally, vascular reactivity studies were performed in third-order branches of rat superior mesenteric arteries, which were obtained and placed in organ baths containing Krebs-Henseleit solution, maintained at 37 °C, gassed with a mixture of 95% O_2_ and 5% CO_2_, and maintained at pH 7.4. The in situ formation of reactive oxygen species (ROS) was evaluated in small mesenteric rings using oxidative fluorescent dihydroethidium dye.

**Results:**

We found higher phenolic content and antioxidant activity in FGS when compared to UGS. HPLC analyses identified a significant number of phenolic compounds with antioxidant potential in both samples. The vasorelaxant effect induced by FGS was more potent than that induced by UGS, and the activity was attenuated after removal of vascular endothelium or by blockade of endothelium-derived relaxing factors, such as NO and EDHF.

**Conclusions:**

The FGS extract may be a great source of natural polyphenol products with potent antioxidant effects and endothelium-dependent vasodilatory actions involving NO and EDHF pathways.

## Background

Around the world and over decades, consumption of grapes and their derivative products, such as juices and wines has grown. The São Francisco Valley region in northeastern Brazil is the second largest producer of refined grapes and red wine in the country [1, 2]. Placed in a semi-arid tropical climate, at 8–9 S (latitude) and around 40 W (longitude), the region has specific features, such as: hot climate, abundant water irrigation/drought, and high light/UV intensity, which severely affect phenolic metabolism and increase grape phenolic content [3, 4]. Chemical contents undergo variations which depend on certain factors, such as the environment and techniques used in the vinification process [5, 6].

Grape pomace, a winemaking byproduct composed of pressed skins, seeds, and stems, has potential health benefits which have been assigned to high polyphenolic compound content; also widely found in red wine. It has become an important economical alternative to wine industry [7–11]. High levels of polyphenolic compounds remain in the skin matrix of grape pomace after enological fermentation, and the use of adequate methods to breakdown the cell wall in grape pomace tissues is indispensable for effective phenolic content extraction [5].

The antioxidant potential of phenolic compounds found in grapes and their byproducts, such as red wine, is responsible for an extensive list of biological activities such as anti-inflammatory and anti-aging effects, and principally, cardioprotective actions [12]. The polyphenolic content of grape byproducts, such as red wine, can be classified into two groups: flavonoids such as, quercetin, catechin, and anthocyanin; and non-flavonoids such as hydroxybenzoic acid, gallic and ellagic acid, the hydroxycinnamates caffeic and caftaric acid, and the stilbenes trans and cis-resveratrol, all present in red wine from Brazil’s São Francisco Valley region [13, 14]. Recently, red wine phenolic compounds from this region have demonstrated marked antioxidant and endothelial-dependent vasodilator effects in rat models of hypertension [15, 16].

In this study, we assessed the phenolic content, in vitro antioxidant activity, and vascular relaxation effects of fermented grape skin extracts from Brazilian northeastern wine grape pomace. This study was performed with a view to exploiting the potential of the grape skins as a source of natural antioxidants with biological vascular activities.

## Methods

### Standards and reagents

Phenylephrine hydrochloride (Sigma, cod. P6126), Acetylcholine chloride (Sigma, cod. A6625), Nω-Nitro-L-arginine methyl ester hydrochloride (Sigma, cod. N5751), charybdotoxin (Sigma, cod. C7802), indomethacin (Sigma, cod. I7378), apamin (Sigma, cod. A1289), Dihydroethidium (Sigma, cod. D7008), 2,2-Diphenyl-1-picrylhydrazyl (Sigma, cod. D9132), Folin-Ciocalteu’s (Sigma, cod. 47,641), ascorbic acid, gallic acid, from Sigma-Aldrich®, 4′,6-Diamidino-2-Phenylindole, Dihydrochloride (DAPI) from Invitrogen Molecular Probes™ (cod. D1306) and Dako Fluorescence Mounting Medium (cod. S3023) Na_2_CO_3_, NaCl, KCl, KH_2_PO_4_, NaHCO_3_, C_6_H_12_O_6_, CaCl_2_, MgSO_4_, ethylenediaminetetraacetic Acid (EDTA), ethanol, methanol, from Vetec®.

### Experimental animals and preparations

Twelve-week old male Wistar rats (*Rattus norvegicus*) weighing around 250–300 g were used in all experimental protocols. The animals were housed in groups of four, and given four days to acclimate to the housing facility. The environmental experimental conditions were a room temperature of 21 °C ± 1 °C, humidity of 60% ± 10%, lighting of 325 lx, and a 12:12 light/dark cycle with lights on at 06:00 and off at 18:00, and the environmental enrichment included bedding. The animals were housed in 410X340X160 mm cages (Beira-Mar-BHG, Brazil) and given access to food and water ad libitum. During housing, the animals’ health status was monitored twice daily. No adverse events were observed. In the present study, twenty rats were used, which were divided into the FGS and UGS groups (*n* = 15 and 5, respectively). The study conformed to the International Guide of Care and the Use of Laboratory Animals, and all experimental protocols were submitted to and approved by the Federal University of Paraíba Ethics Committee on Animal Use (CEUA/UFPB n° 1505/13).

### Grape sample

The grape *Vitis vinifera* (L.) var. Petit Verdot from the São Francisco Valley, and used in the process of wine production was used in our studies. The samples were collected in two stages of the wine production: one type was supplied after separation of the must in the first step of fermentation (fermented grape), and the other type was unfermented grape sampled before the winemaking process. Both samples originated from the São Francisco river valley region in Brazil and were provided by EMBRAPA as collected from the 2011 crop of a local vineyard.

### Samples preparation

The skins from unfermented (UGS) and fermented (FGS) grape pomace, were manually separated from the remainder of the plant material and crushed. The material was submitted to the freeze-drying process and then ground. The particle size was homogenized at lower than 0.71 mm mesh. The homogenized powder was submitted to an extraction procedure by ultrasound with a mixture of ethanol-water (50:50) used as the solvent; the liquid-to-solid ratio was 5 g of dry weight per 100 ml of solvent [17–20]. The resultant extract was dried under vacuum pressure by rotary evaporator and then lyophilized for 24 h under the vacuum pressure of 0.024 mbar (FreeZone 6 LABCONCO®) with prior freezing at −80 °C. The dried samples were stored frozen at −22 °C prior the use in the experiments [21].

### Total Phenolic content

The total phenolic content of the different lyophilized samples was determined using the Folin-Ciocalteu’s (F-C) method, with gallic acid as a standard, in methanol medium. This method was based on the reaction of phenolic compounds with a colorimetric reagent detected spectroscopically at a wavelength of 750 nm [22, 23].

Briefly, an aliquot of 100 μL of each sample, standard, or 95% (*v*/v) methanol blank, was added in duplicate to 2 mL microtubes; adding 200 μL 10% (*v*/v) of F–C reagent and vortexing thoroughly. In the next step, adding 800 μL 700 mM Na_2_CO_3_ to each tube and incubating the assay tubes at room temperature for 2 h; transferring 200 μL of sample, standard or blank from the assay tube to a clear 96-well microplate and reading the absorbance of each well at 750 nm. Solutions of gallic acid (ranging from 0.5 to 1 mg/mL) were analyzed in a similar manner to construct a calibration curve. Each sample was analyzed in triplicate and the total phenolic content was expressed as micrograms of gallic acid equivalent/mg of dry weight (μg GAE/mg DW). Data are expressed as mean ± SEM [13].

### Radical-scavenger activity

The modified DPPH^●^ method performed by Sánchez-Moreno et al. (1999) was used. The standard curve was performed (3.94, 7.89, 11.83, 15.77, 19.71, 23.66, and 27.60 μg/mL of DPPH^●^ in methanol) for the DPPH^●^ (μg/mL) reaction medium concentration, determined by linear regression: A_490nm_ = 0.01116*[DPPH^●^]_t_ – 0.01031, *r* ^*2*^ = 0.99. Aliquots of the sample extract or the positive control (ascorbic acid) were prepared at different concentrations and then added to the DPPH 19.71 μg/mL solution (diluted in methanol). The absorbance at 490 nm was measured until the 30 min steady state to better understand the antioxidant behavior, and to design the protocol for the optimal range [24, 25].

The remaining DPPH^●^ (% DPPH_rem_) percentage was calculated as the ratio between DPPH concentration at each reaction time and DPPH concentration at the initial time (*t* = 0), using the following equation:

$$ \%\mathrm{DPPH}\mathrm{rem}{\frac{\left[ DPPH\bullet \right]}{\left[ DPPH\bullet \right] t=0}} {\ast }100 $$

The percentage of remaining steady state DPPH● against the sample concentration was graphically plotted to obtain the amount of antioxidant sample necessary to decrease the initial DPPH● concentration by 50% (EC_50_). The antioxidant reducing power (ARP), an important antioxidant parameter, was calculated as an inverse of the EC_50_ value, such that the larger ARP value was related to a more efficient antioxidant activity [23, 26].

### Chromatographic determination of Phenolic compounds

Reversed-phase high-performance liquid chromatography (HPLC) method was used to analyze the phenolic compounds present in the samples; using the separation module (LC-20 AT, Shimadzu Corporation, Japan) equipped with a C18 column (Vydac, 218 TP, 250 4.6 mm, 5 μm particle size, Sigma–Aldrich, St. Louis, MO, USA). The samples were eluted with a gradient system consisting of solvent A (2% acetic acid, *v*/v), and solvent B (acetonitrile: methanol, 2:1, *v*/v), used as the mobile phase, at a flow rate of 1 mL/min. The samples (20 μL) were directly injected after filtration through a 0.45 μm membrane filter. The gradient system started from 90% A at 0 min, to 80% A at 10 min, 70% A at 15 min, 60% A at 25 min, 50% A at 30–40 min, 75% A at 42 min, and 90% A at 44 min. A photodiode array detector (Rheodyne, USA) was used, and the peaks of the phenolic compounds were monitored at 280 nm [27]. The Gower similarity coefficient was used to determine relations between the chemical compositions of FGS and UGS, in PAST 3.11 software [28].

### Lyophilized FGS and UGS grape skins extracts induce Vasorelaxation on small mesenteric arteries

Before each experiment, male Wistar rats (wt, 250–300 g) were anesthetized with a pentobarbital sodium (100 mg/kg, i.p.) injection (dissolved in 0.9% sterile saline), and were euthanized by cervical dislocation without suffering. The third-order branches of the superior mesenteric arteries were identified, dissected, and ring segments (1.5–2.0 mm in length), were mounted in the Mulvany apparatus, composed of a small vessel chamber, and a myograph for isometric tension measurements (DMT® myograph 610 M, Aarhus N, Denmark). In certain experiments, the endothelium was removed by rubbing the intima with a single hair [29]. The rings were suspended in organ baths containing Krebs-bicarbonate solution (mM: NaCl 119, KCl 4.7, KH_2_PO_4_ 1.17, MgSO_4_ 1.18, CaCl_2_ 2.5, NaHCO_3_ 25, EDTA 0.027 and D-glucose 5.5; pH 7.4 and 37 °C) and aerated with a mixture of 95% O_2_ and 5% CO_2_.

After an initial equilibration period of 60 min, the integrity of the endothelium was pre-assessed by contracting the tissues with phenylephrine (PE, 1–10 μM) and then adding acetylcholine (1–10 μM). Tissues in which the acetylcholine reversed the phenylephrine-induced tone by more than 90% were designated as endothelium-intact rings and tissues in which acetylcholine caused less than 10% relaxation were designated as endothelium-denuded rings. The vasodilator response induced by the lyophilized grape skins extract, FGS or UGS (10–3000 μg/mL), was evaluated in endothelium-intact or endothelium-denuded mesenteric artery rings pre-contracted with PE 10 μM. In the endothelium-intact rings, L-NAME (100 μM), charybdotoxin (50 nM) plus apamin (50 nM) was added to the organ bath at least 20 min before addition of PE (10 μM). These concentrations were respectively chosen for having been shown to inhibit nitric oxide (NO) and endothelium-derived relaxing factor (EDHF) responses in mesenteric rings [30].

### Determination of vascular oxidative stress

The redox-sensitive fluorescent dye dihydroethidium (DHE) was used to evaluate in situ ROS formation. On the day of vascular reactivity studies, mesenteric artery rings (3 to 4 mm in length) were embedded in OCT compound and frozen in a nitrogen bath for cryostat sections. DHE (2.5 *μ*M) was then applied onto the unfixed 14 μm mesenteric artery cryosections for 30 min at 37 °C in a light-protected humidified chamber to determine in situ formation of ROS [31]. To determine the nature of ROS reductions, the rings were incubated with FGS or UGS for 15 min at 37 °C before adding DHE. Sections were then washed three times and DAPI (Molecular Probes™) was added for 5 min following by further washing (twice) before being mounted in Fluorescence Mounting Medium (DAKO©), and coverslipped. Images were obtained with a Fluorescence Eclipse Ti-U Nikon® microscope. Quantification of the staining levels was performed using NIS-element® software. It is important to note that this experimental protocol was performed only because we had removed and frozen, mesenteric artery parts at −80 °C from the rats whose third branch arteries had been used to evaluate vascular reactivity. Thus, no additional animals were used, reducing the number of animals used in the study.

### Statistical analysis

The minimum number of animals was chosen to allow adequate statistical analysis. Values are expressed as mean ± S.E.M. Statistical significance was determined (when appropriate) by using the student’s T test with GraphPad Prism software, version 6.0 (GraphPad Software Inc., La Jolla, CA, USA). The student’s T test was chosen in accordance with the number of animals per group, ranging from 4 to 7. All phytochemical measurements were carried out in triplicate. Relaxation responses are expressed as a percentage of the phenylephrine contraction effect (at 10 *μ*M). Values of *P* < 0.05 were considered statistically significant.

## Results

### Total phenol content

Considering the total phenolic composition obtained by Folin-Ciocalteu’s (F-C) method, as summarized in the Table 1, we found that FGS (185.53 ± 14.73 μg/mg DW) presented about seven times higher total phenolics than that found in UGS (25.29 ± 0.30 μg/mg of DW).

**Table 1:** Antioxidant reducing power and EC_50_ values obtained in DPPH assay, and total phenolic compounds

Samples	DPPH		TP (μg of GAE / mg of DW)
	*EC*_*50*_ (μg of ext. / μg of DPPH)	*ARP*	
-AA-	0.50 ± 0.05	2.00	-
-FGS-	1.10 ± 0.14 *	0.91	185.53 ± 14.73
-UGS-	1.91 ± 0.42 **^; #^	0.52	25.29 ± 0.30 ^##^

Mean values ± SEM of triplicate are shown. (AA), Ascorbic Acid; (FGS), Fermented Grape Skin; (UGS), Unfermented Grape Skin; (−), Not Analyzed. * significant when compared to AA, *P* < 0.05; ** significant when compared to AA, *P* < 0.01; ^#^ significant when compared to FGS and AA, *P* < 0.05; ^##^ significant when compared to FGS, *P* < 0.05

### DPPH radical scavenger activity

The ARP was evaluated based on an inverse value of EC_50_ which express the amount antioxidant necessary to decrease by 50% the initial DPPH^•^ concentration for each sample [26]. The EC_50_ results showed that the samples respectively presented 0.50 ± 0.05, 1.10 ± 0.14 and 1.91 ± 0.42, for Ascorbic Acid (AA), FGS, and UGS. This relationship between samples confirms the better antioxidant activity (ARP) of FGS, with 0.91, almost twice that of UGS 0.52, and half that of the Ascorbic Acid potential scavenger 2.00 (Table 1).

### HPLC analysis

The HPLC analysis performed with the FGS and UGS extracts revealed the presence of important substances with antioxidant activity and a large content of phenolic compounds and acids. As shown in Table 2, both qualitative and quantitative differences were observed for most of the evaluated sample compounds, such as ferulic acid, sinapic acid, hesperetin and chrysin, which were present only in the fermented samples (FGS). Interestingly, catechin, 2,5-dihydroxybenzoic, and syringic acid are present in higher amounts in FGS compared to UGS (FGS presented twice the catechin and three times the amount of 2,5-dihidroxibenzoic and syringic acid than UGS). It is still important to note that FGS presents the same quercetin content as that found in UGS. None of the samples presented naringenin content (Table 2).

**Table 2:** Phenolic components identified in dried extracts from fermented (FGS) and unfermented (UGS) grape skin

PHENOLIC COMPOUNDS ANALISED	(μg of phenolic compounds / mg of dried extract)	
	FGS	UGS
2,5-dihydroxybenzoic acid	16.8	9.0
2,4-dihydroxybenzoic acid	0.6	0.6
4-hydroxybenzoic acid	0.4	0.4
sinapic acid	0.6	0.0
syringic acid	1.6	0.6
vanillic acid	2.4	2.4
ferulic acid	0.8	0.0
caffeic acid	3.8	4.0
quercetin	2.4	2.2
naringenin	0.0	0.0
catechin	2.6	0.8
hesperetin	0.4	0.0
chrysin	1.0	0.0

### Vasorelaxant-effect induced by lyophilized FGS and UGS skins extract on small rat mesentery artery

**Fig. 1:**
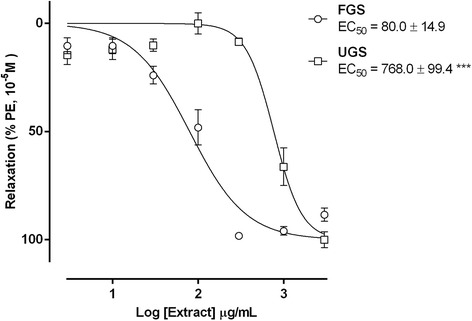
Vasorelaxant effect induced by fermented (FGS) and unfermented (UGS) grape skin extracts (10–3000 μg/mL). The responses were assessed in endothelium-intact mesenteric artery ring resistance pre-contracted to PE 10 μM. Data are expressed as mean ± SEM of 5 and 7 experiments, respectively. *** significant when compared to FGS, *P* < 0.001

In phenylephrine (10 μM) pre-contracted mesenteric resistance artery rings, FGS or UGS (10 to 3000 μg/mL), caused concentration dependent relaxation (Fig. 1) with significantly lower potency in UGS responses as compared to FGS (maximum relaxation = 100.0% ± 3.7%; EC_50_ = 768.0 μg/mL ± 99.4 μg/mL, *n* = 7 and 96.9% ± 4.4%; EC_50_ = 80.0 μg/mL ± 14.9 μg/mL, *n* = 5, respectively).

**Fig. 2:**
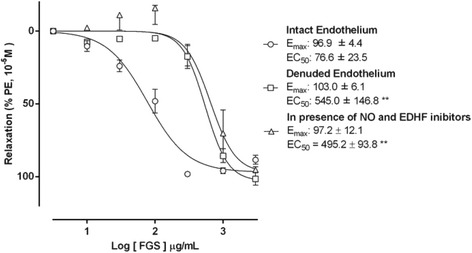
Vasorelaxant effect induced by fermented (FGS) grape skin extracts (10–3000 μg/mL). The responses were assessed in resistance mesenteric artery rings, pre-contracted with PE 10 μM in endothelium-intact, endothelium-denuded and in endothelium-intact preparation, pre-incubated with L-NAME (100 μM) and charybdotoxin (50 nM) plus apamin (50 nM). Data are expressed as mean ± SEM of 5, 4, and 4 experiments, respectively. ** significant when compared to endothelium-intact preparations, *P* < 0.01

The endothelial role in the FGS vasorelaxant effect was evidenced by the response after removal of the vascular endothelium, where FGS elicited vasorelaxation was significantly attenuated (maximum relaxation = 103.0% ± 6.1%; EC_50_ = 545.0 μg/mL ± 146.8 μg/mL; *p* < 0.05; *n* = 4). The endothelial-dependent response was assessed in the presence of L-NAME, to prevent endothelial NO-formation, and in presence of charybdotoxin plus apamin, to inhibit EDHF-mediated responses. In these conditions, the vasorelaxation induced by FGS was significantly attenuated (maximum relaxation = 97.1% ± 12.1%; EC_50_ = 495.2 μg/mL ± 93.8 μg/mL, *n* = 4, Fig. 2) suggesting a strong participation of both, NO and EDHF, in the response induced by FGS.

### Vascular oxidative stress

**Fig. 3:**
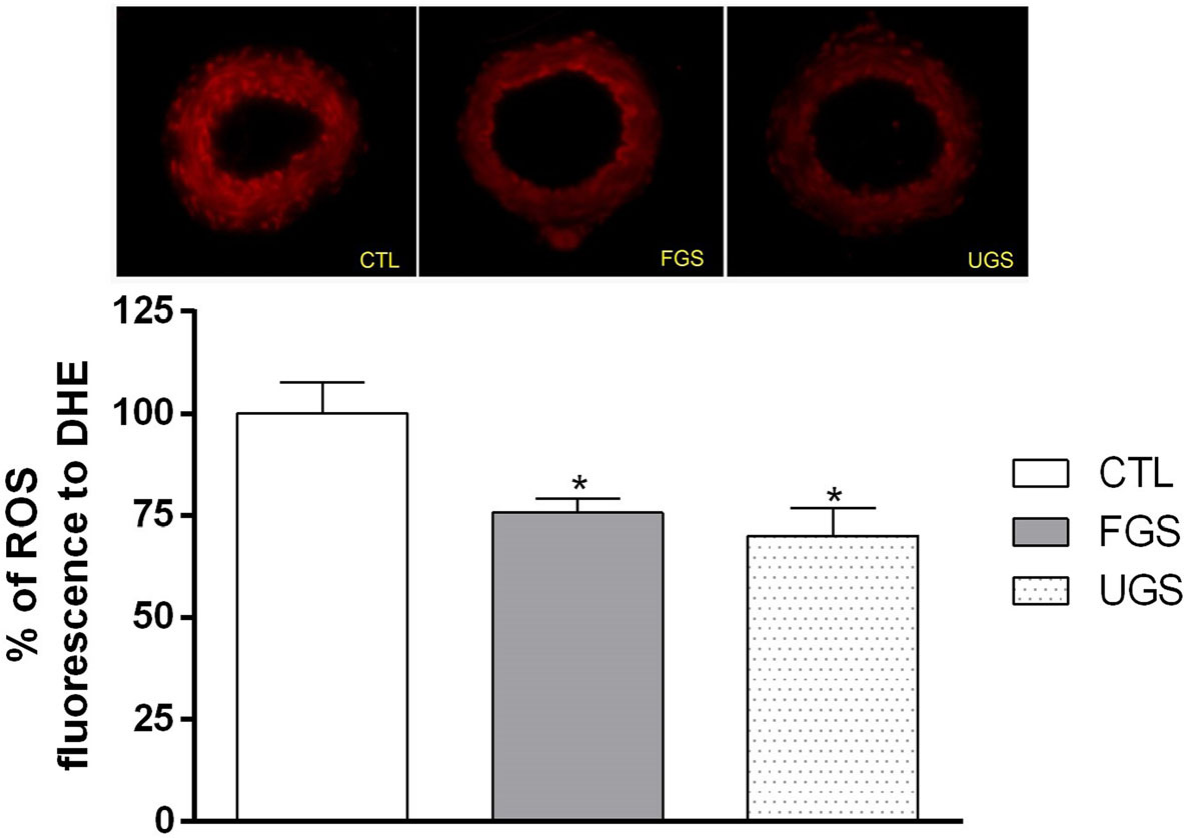
ROS measurement in intact mesenteric arteries exposed to different experimental conditions. Reduced effects of redox-sensitive fluorescence dye to DHE in normotensive small mesenteric artery sections exposed to vehicle (PBS), fermented (FGS) or unfermented (UGS) grape skin extracts. Data are expressed as mean ± SEM of 4 experiments. * significant when compared to controls (CTL), *P* < 0.05

In vitro free radical scavenger activity was confirmed by effects on ROS production in small mesenteric artery rings (*n* = 4). ROS fluorescence to DHE ratio in treated vessels (FGS and UGS) was significantly reduced when compared to artery rings in basal conditions. However, no significant difference was observed between FGS and UGS (Fig. 3).

## Discussion

The presence of phenolic compounds with antioxidant activity in FGS justifies the proposal of an alternative use for fermented extract as a grape byproduct. Fermented grape skins extract is a potential polyphenols-rich source with a diverse phenolic composition, including, catechins, monomeric and oligomeric proanthocyanidins, and glycosylated flavonols [5].

The high content of phenolic compounds provides a basis for the antioxidant activity reported in the DPPH analysis, which like others studies, establishes a positive correlation between antioxidant activity and the total polyphenolic content of the samples [19]. The kinetic behavior of the antioxidant activity for FGS was more salient than for UGS; though both yielded lower potential (intermediated-slow fit curve, time of steady state >30 min) than ascorbic acid (data not showed), which was used as the positive control, and which has rapid kinetic behavior [32]. Similar studies have shown the positive effect of the fermentation process; which yields higher antiradical activity than for unfermented samples [33]. Thus the potent antioxidant activity found in FGS was probably due to the fermentation process, which probably induced chemical changes in the phenolic composition of the extract; as a consequence of pomace cell structure degradation and polyphenol releases [33, 34].

Polyphenols derivatives of benzoic acid, and stilbene such as cis-resveratrol which are found in large amounts in São Francisco Valley region red wines; and flavonoids, such as quercetin, and catechins are closely associated with both antioxidant capacity and beneficial biological effect including cardioprotective actions [35, 36]. For this reason, grape pomace byproducts should be considered a rich potential source of natural phytochemicals that are particularly suitable for use as an ingredient in foods, medicines, and cosmetics. Taken together, our observations provide evidence that its potential effects are related to the presence of a greater number of polyphenolic compounds in FGS.

The experimental protocols of the present study were performed in rats since, to our knowledge; there are no alternative techniques to evaluate the effects of a product on vascular reactivity that do not involve the use of animal models or humans beings. Thus, to investigate the vasorelaxant effect elicited by FGS and UGS, endothelium-intact rat mesenteric artery rings were pre-contracted with phenylephrine (1 μM), an α1-adrenoceptor agonist. In the presence of this contracting agent, FGS and UGS induced significant and concentration-dependent relaxation. Interestingly, FGS-induced vasorelaxation was about 10 times more potent than that induced by UGS. Considering this strong difference between FGS and UGS, and in order to diminish the number of animals used in the present study, we decided to elucidate only the FGS mechanism for inducement of vasorelaxant activity. FGS induced concentration-dependent relaxation, with increased potency in the presence of vascular endothelium. In mesenteric rings pre-incubated with selective inhibitors of the NO and EDHF pathways, FGS induced a similar response to that presented for preparations in the absence of endothelium. These results strongly suggest that the mechanism by which these effects occur seems to be dependent on endothelium-derivative relaxant factors such as NO and EDHF. The data demonstrates the importance of these two pathways in the endothelial response induced by FGS. Indeed, the improvement of vascular tone by grape polyphenols, induced by the formation of NO and stimulation of EDHF pathways is well established [37]. The results obtained so far show consistently that the polyphenols in FGS have the capacity to improve endothelial control of vascular tone; not only in a specific rat experimental model but also in healthy and diseased humans. [38, 39].

Finally, for FGS, we found marked induced tissue antioxidant effect. These effects on ROS reduction are relevant to several disorders such as hypertension, which is related to an increase in the production of the superoxide anion and hydrogen peroxide, reduced nitric oxide synthesis, and decreased the bioavailability of antioxidants [36]. As a matter of fact, therapies targeted at decreasing ROS generation or increasing nitric oxide availability may be useful for minimizing vascular injury and renal dysfunction, thereby preventing or reducing end-organ hypertensive damage [40, 41].

## Conclusions

The results obtained show that the radical scavenger effect induced by fermented as compared to unfermented grape skins might be explained, by the higher content of total phenols presented, which itself may be explained by the fermentative procedure in the first step of the winemaking process. Grape skin in pomace presents high phenolic content; being rich in anthocyanins, benzoic and hydroxycinnamic acids, catechins, flavonol glycosides, phenols acids and alcohols, stilbenes. Considered to have biological properties, these compounds are the main candidates. Indeed, total phenol content is strongly correlated with the antioxidant and vasorelaxant activities. Thus, FGS might be of great interest for its antiradical/antioxidant and endothelium-dependent vasodilator actions, inducing vasodilation that may involve NO and EDHF pathways.

Taken together, the results obtained so far suggest that both the components and biological activities described in this work support the proposed use of grape byproducts, obtained from the wine production process, as a potential source of polyphenolic compounds for use in the food industry (nutraceutical, and/or pharmaceutical industries). The data obtained here, on a laboratory-scale, may be useful in the development of industrial scale FGS processing.

## Abbreviations

AA: ascorbic acid
ARP: antioxidant reducing power
DAPI: 4′,6-Diamidino-2-Phenylindole, dihydrochloride
DHE: dihydroethidium
DPPH: 2,2-Diphenyl-1-Picrylhydrazyl
EDHF: endothelium-derived hyperpolarizing factor
EDTA: ethylenediaminetetraacetic acid
F-C: Folin-Ciocalteu’s
FGS: fermented grape pomace
HPLC: reversed-phase high performance liquid chromatography
L-NAME: Nω-Nitro-L-Arginine methyl ester hydrochloride
NO: nitric oxide
PE: phenylephrine
ROS: reactive oxygen species
UGS: unfermented grape pomace
